# Human metapneumovirus - what we know now

**DOI:** 10.12688/f1000research.12625.1

**Published:** 2018-02-01

**Authors:** Nazly Shafagati, John Williams

**Affiliations:** 1 Department of Pediatrics, University of Pittsburgh School of Medicine, Pittsburgh, PA, USA

**Keywords:** human metapneumovirus, acute respiratory infection, Viral pneumonia

## Abstract

Human metapneumovirus (HMPV) is a leading cause of acute respiratory infection, particularly in children, immunocompromised patients, and the elderly. HMPV, which is closely related to avian metapneumovirus subtype C, has circulated for at least 65 years, and nearly every child will be infected with HMPV by the age of 5. However, immunity is incomplete, and re-infections occur throughout adult life. Symptoms are similar to those of other respiratory viral infections, ranging from mild (cough, rhinorrhea, and fever) to more severe (bronchiolitis and pneumonia). The preferred method for diagnosis is reverse transcription-polymerase chain reaction as HMPV is difficult to culture. Although there have been many advances made in the past 16 years since its discovery, there are still no US Food and Drug Administration-approved antivirals or vaccines available to treat HMPV. Both small animal and non-human primate models have been established for the study of HMPV. This review will focus on the epidemiology, transmission, and clinical manifestations in humans as well as the animal models of HMPV pathogenesis and host immune response.

## Introduction

The start of the twenty-first century has seen the discovery of several emerging or new respiratory pathogens causing human disease, including severe acute respiratory syndrome coronavirus and human metapneumovirus (HMPV). The metapneumoviruses are enveloped, non-segmented, negative-sense, single-stranded RNA viruses ^1^. They comprise a genus of two species: avian metapneumovirus and HMPV. The metapneumoviruses belong to the order *Mononegavirales* and family *Pneumoviridae*, which also includes respiratory syncytial virus (RSV) ^2, 3^.

## Avian metapneumovirus

Avian metapneumovirus (previously known as turkey rhinotracheitis virus) was discovered in 1978 in turkeys in South Africa ^4^. Since then, the virus has been recognized to infect turkeys, chickens, and ducks worldwide with a significant economic impact ^5^. The virus has a low and variable mortality but high morbidity rate (up to 100%) and causes severe upper respiratory infections as well reproductive issues leading to decreased egg production ^5^. There are currently four subtypes of avian metapneumovirus based on the genetic diversity of the attachment (G) protein ^6^. Subtype A was first isolated in South Africa, followed by subtype B in several European countries. Subtype C was discovered in the US in 1996 ^7^, and subtype D was identified in France in 2000 ^8^. It is thought that wild migratory birds play a key role in the spread of avian metapneumovirus ^5^.

## Discovery of human metapneumovirus

In 2001, researchers in the Netherlands first identified HMPV from stored nasopharyngeal samples from 28 children with respiratory illness by using electron microscopy and random reverse transcription-polymerase chain reaction (RT-PCR) techniques. This novel virus exhibited cytopathic effect but not hemadsorption in tertiary monkey kidney epithelial cells. The genome was most closely related to avian metapneumovirus serotype C (up to 88% homology). However the newly discovered virus replicated efficiently in monkeys but not in birds ^2^. Archived sera from the 1950s contained neutralizing antibodies against HMPV ^2^. Two retrospective Canadian studies detected HMPV in specimens collected from patients with respiratory illness between 1993 and 2001 ^9, 10^, and a US study detected HMPV in specimens from 1976 to 2001 ^11^. Collectively, these studies show that HMPV has been circulating undetected for many decades.

## Genome organization and structure

HMPV is a negative-sense, non-segmented, single-stranded RNA virus. The genome is about 13,000 nucleotides in length and is composed of eight genes encoding for nine proteins: nucleoprotein (N), phosphoprotein (P), matrix protein (M), fusion protein (F), matrix-2 proteins (M2-1 and M2-2), small hydrophobic (SH) protein, glycoprotein (G), and large (L) polymerase protein ( Table 1) ^12– 21^. As in other paramyxoviruses, the N, L, and P proteins form the viral replication complex. Though similar in genome to RSV, both avian metapneumovirus and HMPV possess a gene order different from that of RSV and lack the non-structural proteins NS1 and NS2 ^1^. HMPV exhibits a paramyxovirus-like morphology, ranging from 150 to 600 nm in size, enveloped with short protein spike projections ^2^.

**Table 1.** Summary of human metapneumovirus proteins and function.

Gene	Protein	Amino acid length	Function
N	Nucleoprotein	394	RNA genome encapsidation
P	Phosphoprotein	294	Polymerase co-factor
M	Matrix protein	254	Aids in viral assembly and budding
F	Fusion protein	539	Virus-cell binding and membrane fusion
M2	M2-1 protein	187	RNS transcription processivity factor
	M2-2 protein	71	Regulates RNA transcription/replication
SH	Small hydrophobic protein	177–183	Possible viroporin or innate immune inhibition
G	Attachment glycoprotein	229–236	Binds to cellular glycosaminoglycans
L	Large polymerase protein	2,005	Catalytic activity for viral replication

Several respiratory viruses form filamentous viral particles *in vitro*. A recent study demonstrated that HMPV infection led to the formation of branched viral filamentous networks and intercellular extensions in human bronchial epithelial cells ^22^. HMPV P protein co-localized with actin and induced the formation of the intercellular extensions. Importantly, HMPV spread directly from cell to cell even in the presence of neutralizing antibodies and in the absence of the attachment factor heparan sulfate. This direct viral spread was mediated by the actin cytoskeleton, CDc42, and Rac1 ^22^.

## Viral replication

Replication of HMPV occurs in the nasal and lung tissues, and airway epithelial cells are the primary target of HMPV. HMPV is thought to attach to the target cell via G protein interactions with heparan sulfate and other glycosaminoglycans ^21, 23^. The HMPV F protein encodes an RGD (Arg-Gly-Asp) motif that engages RGD-binding integrins as cellular receptors ^16, 24, 25^ and then mediates fusion of the cell membrane and viral envelope in a pH-independent fashion, likely within endosomes ^5, 26– 28^. Low pH-dependent fusion is a rare occurrence in only certain lineage A F proteins ^29^. As with other negative-sense, single-stranded RNA viruses, HMPV negative-sense genomic RNA is transcribed to positive-sense mRNA by the RNA polymerase before translation. After translation, the viral glycoproteins are transported from the endoplasmic reticulum through the Golgi apparatus to the plasma membrane. As nascent viral proteins accumulate, the polymerase switches from transcribing monocistronic mRNA to replicating full-length positive-sense antigenome to serve as a template for progeny negative-sense genomes. Newly synthesized virions exit the host via budding from the plasma membrane, which is facilitated by the M protein ^30, 31^.

## Phylogenetic groups

There are two major genetic lineages, A and B, further divided into the sublineages (or clades) A1, A2, B1, and B2 ^32– 35^. All HMPV genes fall into these four clades, suggesting that genome recombination is very rare ^33^. Although both genotypes A and B can co-circulate, the dominant lineage may vary year by year ^33, 36^. Phylogenetic analyses of HMPV suggest that the human virus diverged from the avian type C 200–400 years ago ^33– 35, 37^. HMPV cannot productively infect birds; thus, if HMPV did arise in humans as a zoonotic infection, the virus is now adapted fully to humans ^2^. Evidence suggests that the different genetic lineages of metapneumovirus do not represent distinct serotypes; studies in rodents and non-human primates show a high degree of cross-neutralization and cross-protection between subgroups ^38– 41^.

## Epidemiology

HMPV has a seasonal distribution that is similar to that of other respiratory viruses and tends to peak in later months compared with RSV and influenza ^11, 41– 45^. Similar to other respiratory pathogens, HMPV causes most severe disease in infants and young children, the elderly, and persons with underlying chronic conditions such as asthma, emphysema, and immune compromise. Seroepidemiology studies have shown that most children worldwide are infected with HMPV by the age of 5 ^2, 46– 50^.

### Children

Rates of hospitalization of children for HMPV infection are highest in the first year of life but occur throughout early childhood. Many studies report that the peak age of hospitalization for HMPV is between 6 and 12 months of age, which is later than the peak age of hospitalization for RSV (2–3 months) ^44, 51– 56^. Through the course of a year, multiple subgroups of HMPV will often be in circulation. These subgroups are genetically distinct and can vary across seasons, with one lineage being more prevalent in a given season ^45, 57^. Despite genetic differences between the subgroups, all remain capable of causing severe infection, and the differences have not been associated consistently with the variation in severity of disease ^58, 59^. HMPV infection usually causes overt disease; the virus is rarely detected in asymptomatic children ^11, 44, 60– 63^. Studies of hospitalized and outpatient children worldwide have found HMPV to be associated with 6 to 40% of acute respiratory illness ^11, 41, 44– 46, 51– 53, 64– 67^.

### Adults

Although nearly all populations will experience primary HMPV infection by age 5, HMPV re-infection occurs throughout adult life. HMPV was identified in up to 13% of hospitalized adults in Rochester, New York ^43^. Whereas HMPV infection is typically mild in otherwise healthy younger adults, infection with HMPV leads to increased disease severity and high morbidity and mortality rates in the elderly. A retrospective Canadian study showed that 46% of HMPV ^+^ cases were from patients at least 65 years, and 60% of these elderly patients were hospitalized ^10^. A subsequent study reported that at least 50% of the HMPV ^+^ elderly patients infected during an outbreak in a long-term care facility developed bronchitis or pneumonia, leading to 50% mortality ^68^.

### Underlying conditions

Pre-existing conditions, particularly asthma, play a role in disease severity and hospitalization. HMPV was isolated from 7% of adults hospitalized for an acute asthma exacerbation ^69^. Like infection with RSV, infection with HMPV within the first two years of life is a risk factor for later asthma ^70^. One study found that 16% of HMPV ^+^ patients had asthma compared with none of the RSV ^+^ patients ^54^. Another study noted a previous asthma diagnosis in 41% of HMPV ^+^ children between the ages of 5 and 13 ^42^.

Immunocompromised patients and those with underlying medical conditions can be severely affected by HMPV. One study found that many HMPV ^+^ hospitalized patients over the age of 5 had other severe diseases, such as cystic fibrosis or lymphoma ^54^. Another retrospective study found that of 39 immunocompromised children with HMPV, 17 developed pneumonia and four died from respiratory failure ^71^. In a group of patients between the age of 15 and 65 years, 67% had underlying medical conditions, such as lymphoma or lung tumors ^10^.

Co-infections with other viral or bacterial pathogens may exacerbate symptoms and disease. Viral co-infection rates in patients with HMPV range from 6 to 23% ^11, 42, 43^, but viral co-infections do not seem to impact disease severity ^11^. However, secondary bacterial pneumonia can occur and is associated with increased mortality ^72– 74^.

## Transmission and symptoms

HMPV is thought to spread through direct or close contact with infected individuals or objects (fomites) ^36^. Symptoms and disease presentation of HMPV are similar to those of other respiratory viruses causing both upper and lower respiratory tract infections. Symptoms can include cough, rhinorrhea, sore throat, and fever as well as lower respiratory tract symptoms such as wheezing, difficulty breathing, and hypoxia ^54, 75^. The clinical diagnoses most commonly associated with HMPV are bronchiolitis and pneumonia ^42^.

## Animal models

Although early studies demonstrated that HMPV does not replicate or cause disease in birds ^2^, small animal models such as mice, cotton rats, and hamsters as well as non-human primates are semi-permissive ^40^. Several studies show that cotton rats are the most permissive small animal model and that peak virus titers occurred at day four post infection ^76– 78^. Viral lung replication and disease vary between different inbred mouse strains; most work has been published in the BALB/c model, which exhibits substantial disease symptoms ^79– 88^. BALB/c and C57BL/6 mice may exhibit clinical symptoms such as difficulty breathing, weight loss, and ruffled fur, partly depending on the virus strain and inoculum ^80, 83, 89^. Histological scoring revealed that lung pathology is most severe between days 5 and 7 but is significantly decreased by day 14. Viral replication occurs for up to 10–14 days in mice, and peak viral load is at day 5 ^80, 83, 89^. Similar to older humans, aged mice have increased disease severity, higher viral titers, and diminished immune response compared with younger mice ^90^. However, unlike humans, in whom re-infection occurs throughout life, immunocompetent mice cannot be productively re-infected with HMPV. Of note, most work has been published in the BALB/c inbred strain, based on the extensive body of RSV research, but some investigators have focused on the C57BL/6 model.

In contrast, cotton rats, hamsters, and ferrets infected with HMPV do not manifest observable clinical symptoms ^2, 40, 83, 91^. In hamsters and ferrets, there is high viral replication in the respiratory tract compared with mice ^40^. Both African green monkeys and rhesus macaques are permissive for HMPV infection, but neither exhibits clinical symptoms. HMPV replication and neutralizing antibody production are higher in African green monkeys compared with rhesus macaques ^40, 92^.

## Pathogenesis and immunity

Humans and animals mount neutralizing antibody responses to HMPV. In mice, neutralizing antibodies are first detected five to seven days after infection, peaking between four and six weeks after infection. In mouse models, initial infection with HMPV protects against re-infection ^40, 80^, and antibodies alone can protect in small animal models ^93– 96^. In contrast, when macaques were challenged 12 weeks after primary HMPV infection, virus replication was detectable despite the presence of serum antibodies, and when challenged 8 months after primary infection, there was no protection ^97^. These data suggest that in primates and humans, antibody levels wane over time, facilitating re-infection. A prospective study in humans noted that baseline HMPV antibodies were lower in older adults who subsequently became infected versus those who did not become infected ^98^, suggesting a protective effect of antibodies.

Cytotoxic T lymphocytes (CTLs) contribute to clearance of HMPV infection in mice ^80, 89, 99– 101^. As early as day 1 post infection, there is an infiltration of lymphocytes, monocytes, and other mononuclear cells to perivascular and peribronchial areas of the lung. There is an increase in the number of total bronchoalveoloar lavage cells that starts at day 1, peaks at day 7, and returns to near normal numbers by day 21 ^80^. The number of neutrophils and mononuclear cells increases by day 3 until day 14 post infection. CD4 ^+^ T cells peak earlier at day 6 whereas CD8 ^+^ T cells peak between day 8 and 10 ^80, 89, 100^. Furthermore, the depletion of T cells leads to prolonged viral replication ^80, 99, 101^, and T-cell epitope vaccination alone can reduce viral titers ^89, 100^. The role of natural killer (NK) cells is unclear. One study depleted NK cells with anti-CD49 antibody and reported prolonged viral replication ^99^, whereas another depleted NK cells with the more specific antibody anti-NK1.1 and found no effect ^88^.

Although T cells play a critical role in disease protection, they also contribute to disease severity caused by HMPV. The depletion of either CD4 ^+^ or CD8 ^+^ T cells led to significantly less weight loss, decreased lung inflammation, and reduced airway obstruction ^80^. These results collectively show that T cells, especially CD4 ^+^ T cells, play a role in enhancing clinical disease and lung pathology.

HMPV, like other respiratory viruses, dampens the immune response after infection, but the specific mechanisms remain unclear. Multiple studies demonstrate convincingly that HMPV can interfere with the type I interferon (IFN) response, but different studies have implicated various viral proteins, including G, M2-2, P, or SH, and suggested diverse mechanisms ^19, 102– 105^. Type I IFN receptor (IFNAR)-deficient mice infected with HMPV had higher lung viral titers than wild-type (WT) mice but less lung inflammation and airway dysfunction, highlighting the importance of the IFN pathway for HMPV clearance and disease ^86^. HMPV is capable of infecting human and murine dendritic cells *in vitro*, leading to altered signaling, diminished cytokine production, decreased migration, and reduced capacity to activate CD4 ^+^ T cells ^106– 112^. However, the contribution of these interactions to disease and protection *in vivo* has not been defined.

One way that HMPV evades the adaptive immune response is through the upregulation of programmed cell death-1 (PD-1), a T-cell surface receptor that plays a critical role in downregulating the immune response, leading to CD8 ^+^ T-cell functional impairment. This phenomenon is similar to CD8 ^+^ T-cell exhaustion described in chronic infections and cancer ^113^. During infection with HPMV and other acute respiratory viruses, there is an upregulation of both PD-1 and its ligand, PD-L1, in the lungs but not splenic CD8 ^+^ T cells. Blocking PD-1 ligation prevented functional impairment of HMPV-specific CD8 ^+^ T cells in the lung, and mice lacking PD-1 had a greater percentage of functional HMPV-specific CD8 ^+^ T cells compared with WT mice ^89^. During secondary HMPV infection, lung CD8 ^+^ T-cell effector functions were severely impaired after re-infection and PD-1 expression was high; blockade of PD-1 ligation enhanced CD8 ^+^ T-cell function ^100^. These results collectively suggest that the PD-1/PD-L1 pathway plays an important role in evading the immune response during primary and secondary HMPV infections and may contribute to re-infection.

## Diagnosis

The standard method for HMPV diagnosis has been nucleic acid amplification tests, such as RT-PCR ^114– 116^. Several commercial multiplex molecular assays that include HMPV are available ^117^. Viral culture and serological testing are insensitive ^2^. One reason for the delayed discovery of HMPV is the difficulty of growing the virus in cell culture. The virus requires exogenous trypsin to replicate *in vitro* and while capable of growth in other cell lines, it produces robust cytopathic effects in tertiary monkey kidney and LLC-MK2 (rhesus kidney) cells ^2, 118^. Furthermore, viral propagation can take 14 days or longer.

## Antiviral treatments

Treatment consists of supportive care as there are no licensed antivirals against HMPV. Two potential treatments that have been investigated are ribavirin and immunoglobulin. Ribavarin is a nucleoside with activity against RNA viruses and exhibits *in vitro* activity against HMPV ^119^ and exhibited some efficacy in mice ^120^. Commercial intravenous immunoglobulin (IVIG) contains neutralizing activity against HMPV ^119^, and as noted above, antibodies alone exhibit efficacy both prophylactically and therapeutically in mice ^93– 96^. There are anecdotal reports of human use of ribavirin and IVIG ^121^ but no controlled trials and no guidelines to recommend the use of these measures.

## Vaccine development

There are currently no licensed vaccines for HMPV, but numerous efforts have been made to develop a safe and effective vaccine. Early cross-challenge studies with hamsters showed that infection with subgroup A produced an immune response that protected from a subsequent challenge with subgroup B and vice versa ^40^.

There have been several promising live-attenuated vaccines. A cold-adapted, live-attenuated HMPV vaccine provided complete protection in hamsters ^122^. While antibody levels were increased after immunization in cynomolgus macaques, immunization did not provide complete protection from viral replication after challenge ^123^. Recombinant HMPV (rHMPV) viruses lacking the G, M2-1, M2-2, or SH protein have exhibited an attenuated and immunogenic phenotype in animal models ^15, 124, 125^. Mutations in the methyl transferase domains of the polymerase or the integrin-binding RGD motif of the F protein were attenuated, immunogenic, and protective in cotton rats ^126, 127^.

Vectored vaccine approaches that have been effective in animal models include chimeric rHMPV containing the avian metapneumovirus P protein ^128^, alphavirus-vectored HMPV F ^129, 130^, bovine PIV3 vectored F ^39^, or Sendai virus vectored F ^131^. The establishment of a human challenge model ^132^ and a successful test of a live-attenuated candidate in seropositive adults ^133^ provides a platform for future clinical trials.

Another method of vaccination is with heat-killed or formalin-inactivated virus, but a major concern for non-replicating HMPV vaccines is the experience in the 1960s with formalin-inactivated RSV (FI-RSV) vaccines ^134– 136^. FI-RSV induced an aberrant immune response that failed to protect and led to enhanced respiratory disease in vaccinees upon natural RSV infection. Animal studies replicated the results of the FI-RSV clinical trials ^137, 138^. Similar to FI-RSV, FI-HMPV and heat-inactivated HMPV vaccines in mice, cotton rats, and macaques led to enhanced disease following viral infection and to high mortality and increased levels of cytokines and lung inflammation ^139– 141^. These studies show that these kinds of vaccines not only do not induce protective immunity but may lead to increased morbidity and mortality.

In comparison with inactivated vaccines, subunit vaccines that contain partial or full-length viral proteins, particularly the HMPV F protein, have been more encouraging. Several studies of recombinant F protein report protective immunity without enhanced disease in cotton rats, hamsters, and non-human primates ^91, 123^. Another non-replicating vaccine approach is virus-like particles using HMPV M and F proteins expressed in human embryonic kidney epithelial cells ^101, 123, 142^ or generated using retroviral vectors ^143^. All of these approaches have induced neutralizing antibodies and in some cases functional T-cell responses ^101, 142^, leading to protection without signs of enhanced disease. Unfortunately, although subunit vaccines do induce immune responses when challenged with HMPV, the immune response may rapidly wane over time and may require multiple immunizations.

CTL epitopes have been employed as peptide vaccines in mice. CTL epitope vaccines protected mice from HMPV infection by reducing viral load and lung immunopathology, generating effector and memory T-cell response, enhancing T helper 1 (Th1)-type cytokine expression, and reducing Th2-type cytokine expression ^144^. Other peptide vaccine approaches have induced functional memory CD8 ^+^ T cells that were associated with reduced viral titers ^89, 100, 142^. A recent study constructed a multi-epitope peptide (MEP), consisting of six B-cell epitopes, four CTL epitopes, and two T helper cell epitopes. MEP caused both strong humoral immunity, as indicated by increased antibody levels, and cell-mediated immunity, as indicated by increased lymphocyte levels and activity ^145^.

## Future directions

Although HMPV was only discovered in 2001, there have been many advances in understanding mechanisms by which HMPV causes disease. Serologic and evolutionary studies indicate that HMPV has circulated for many years undetected. Robust animal models have been established, and candidate vaccines and antibodies have been developed. However, there is still much in the field regarding pathogenesis, immunity, antivirals, and vaccines that is yet to be discovered.
